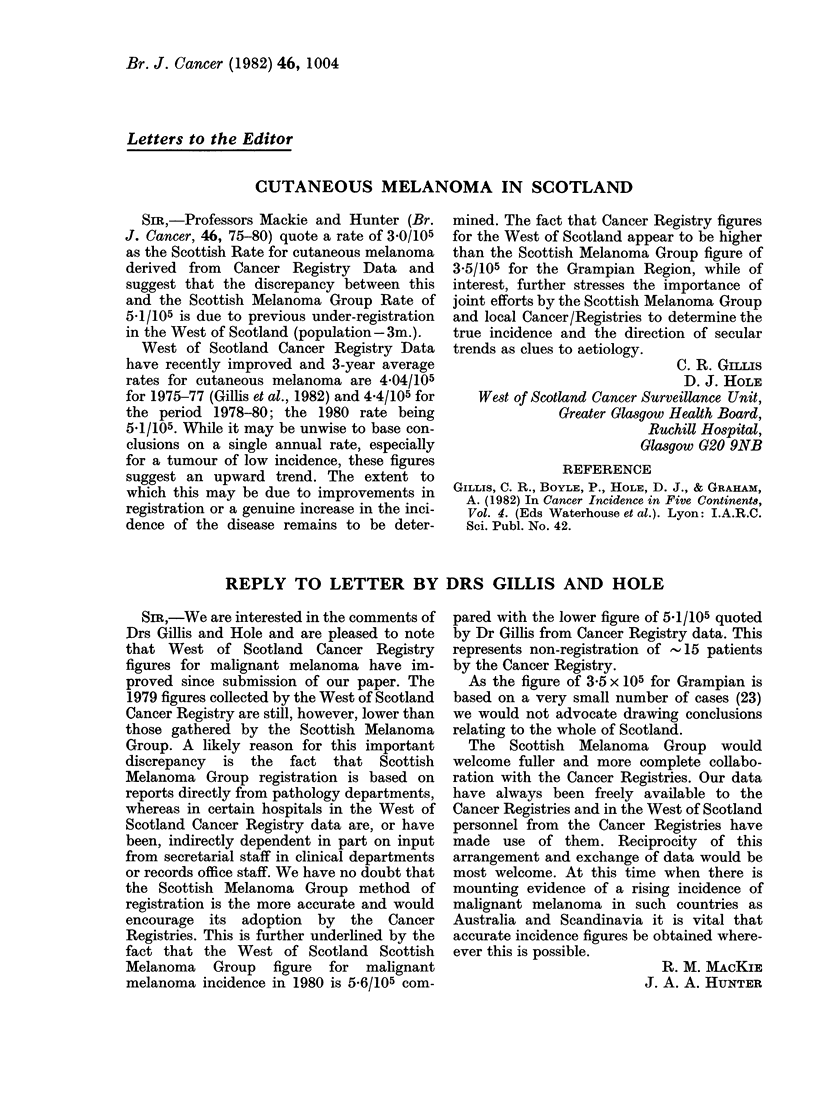# Reply to letter by Drs Gillis and Hole

**Published:** 1982-12

**Authors:** R. M. MacKie, J. A. A. Hunter


### REPLY TO LETTER BY DRS GILLIS AND HOLE

SIR,—We are interested in the comments of Drs Gillis and Hole and are pleased to note that West of Scotland Cancer Registry figures for malignant melanoma have improved since submission of our paper. The 1979 figures collected by the West of Scotland Cancer Registry are still, however, lower than those gathered by the Scottish Melanoma Group. A likely reason for this important discrepancy is the fact that Scottish Melanoma Group registration is based on reports directly from pathology departments, whereas in certain hospitals in the West of Scotland Cancer Registry data are, or have been, indirectly dependent in part on input from secretarial staff in clinical departments or records office staff. We have no doubt that the Scottish Melanoma Group method of registration is the more accurate and would encourage its adoption by the Cancer Registries. This is further underlined by the fact that the West of Scotland Scottish Melanoma Group figure for malignant melanoma incidence in 1980 is $5{\cdot}6/10^5$ compared with the lower figure of $5{\cdot}1/10^5$ quoted by Dr Gillis from Cancer Registry data. This represents non-registration of $\sim 15$ patients by the Cancer Registry.

As the figure of $3{\cdot}5 \times 10^5$ for Grampian is based on a very small number of cases (23) we would not advocate drawing conclusions relating to the whole of Scotland.

The Scottish Melanoma Group would welcome fuller and more complete collaboration with the Cancer Registries. Our data have always been freely available to the Cancer Registries and in the West of Scotland personnel from the Cancer Registries have made use of them. Reciprocity of this arrangement and exchange of data would be most welcome. At this time when there is mounting evidence of a rising incidence of malignant melanoma in such countries as Australia and Scandinavia it is vital that accurate incidence figures be obtained wherever this is possible.

R. M. MACKIE
J. A. A. HUNTER